# Baseline vagal tone and pain reactivity: linking autonomic function to endogenous pain inhibition in women

**DOI:** 10.1097/PR9.0000000000001390

**Published:** 2026-02-09

**Authors:** Einav Gozansky, Hadas Okon-Singer, Irit Weissman-Fogel

**Affiliations:** a Department of Psychology, School of Psychological Sciences, University of Haifa, Haifa, Israel; b The Integrated Brain and Behavior Research Center (IBBR), University of Haifa, Haifa, Israel; c Artificial Intelligence Research Center (HiAI), University of Haifa, Haifa, Israel; d Physical Therapy Department, Faculty of Social Welfare and Health Sciences, University of Haifa, Haifa, Israel

**Keywords:** Pain inhibition, Vagal tone, Vagal reactivity, Menstrual cycle, Healthy females

## Abstract

**Introduction:**

Conditioned pain modulation (CPM) is a laboratory test of the efficacy of endogenous pain inhibition. Although the vagus nerve is thought to contribute to analgesia via descending inhibitory pathways, its role in CPM remains unclear. In addition, as vagal tone fluctuates across the menstrual cycle due to hormonal changes, the menstrual cycle's influence on the vagal–CPM association warrants investigation.

**Methods:**

Sixty-seven healthy females completed psychological questionnaires and underwent psychophysical assessments (1-minute tonic heat pain [THP] given alone and during CPM). An electrocardiogram was recorded at rest, and in 1-minute segments before, during, and after the THP alone, as well as during a conditioning stimulus (CPM paradigm). Heart rate variability was assessed using time- and frequency-domain indices. Menstrual phase was categorized as either follicular or luteal based on standard cycle-length division.

**Results:**

The interaction between the resting root mean square of successive differences (RMSSD) and the menstrual cycle predicted better CPM efficiency. Post-hoc analysis revealed that the vagal–CPM association emerged solely in the follicular phase (β = −1.05, *P* = 0.010). Nevertheless, psychological factors did not influence the vagal–CPM association. The RMSSD broadly changed under pain with conditioning compared to baseline, with higher levels of RMSSD positively associated with CPM magnitude.

**Conclusions:**

The findings suggest a tentative menstrual cycle–dependent association between baseline vagal tone and pain inhibition in healthy females. Further, greater vagal activity contributes to more efficient pain inhibition. Future studies should use within-subject designs to examine how interactions between vagal and sympathetic activity across the menstrual cycle influence pain modulation.

## Abstract 2

Higher baseline vagal tone in the follicular phase and greater vagal activity during heat pain with conditioning (conditioned pain modulation) predict more efficient pain inhibition.

## 1. Introduction

The pain-inhibitory role of the vagus is known based on animal studies^56,62^ and human studies using vagal nerve stimulation.^13,48,58^ In humans, vagal afferents originating from baroreceptors project centrally to the nucleus of the solitary tract,^8^ which connects to the parabrachial nucleus, limbic structures (eg, amygdala, hypothalamus), and the periaqueductal gray—a key structure in the descending inhibitory pathways.^7^ These neural connections modulate pain through both autonomic control and spinal nociceptive inhibition, and by influencing emotional responses to noxious stimuli.^15,69^ In addition, vagal afferents influence somatic nociceptive transmission in the spinal dorsal horn and modulate peripheral inflammation, contributing to pain regulation through both central and peripheral mechanisms.^9,15^

The function of the descending inhibitory pathways in humans is commonly evaluated using the conditioned pain modulation (CPM) paradigm, which is based on the pain-inhibiting-pain phenomenon.^31,55^ In this paradigm, pain ratings in response to a test stimulus are modulated by a conditioning painful stimulus applied in a remote area. While the mechanisms underlying CPM have been extensively studied, the role of the vagus nerve in CPM efficiency remains poorly understood, with evidence showing inconsistent effects that appear to depend on physiological and psychological factors such as body composition, affective state, and sex.^3,25,73^ For instance, while one study reported a positive association between vagal tone and CPM in males but not females,^49^ others found this relationship only in individuals with low-to-moderate physical activity levels,^46^ or specifically in females with elevated anxiety.^51^

Complementary evidence from animal models highlights sex-specific mechanisms in vagal pain modulation.^32,33,82^ For example, in male rodents, vagal tone reduction suppressed pain only when gonads were intact, but was unrelated to androgen levels. In contrast, in females, vagal influences on pain were contingent on both gonadal status and estrogen levels.^32^ Consistent with these findings, hormonal fluctuations across the menstrual cycle influence vagal tone in women, with a systematic review showing a decline in vagal tone from the follicular to the luteal phase as estrogen levels decrease.^65^ These hormone-related shifts in autonomic regulation may contribute to menstrual phase–dependent differences in the efficiency of CPM, as suggested by some studies,^63,75^ although others find no consistent influence on pain.^27,79,81^ Collectively, these findings underscore the importance of accounting for the hormonal menstrual cycle phase when investigating the contribution of vagal tone to pain modulation in females, which has not been directly examined.

Psychological variables, such as depression and pain catastrophizing, have also been linked to individual differences in CPM magnitude.^50,51,53,77^ Further, vagal tone influences mood and emotional regulation, with vagus nerve stimulation alleviates anxiety and depression in psychiatric and pain populations.^57,84^ Therefore, it is assumed that psychological factors may mediate the vagal–CPM relationship. Supporting this notion, a previous study suggests that changes in psychological distress partially mediate the analgesic effects of the vagus.^20^

Although resting vagal tone has received considerable attention, only one study focused on vagal reactivity during CPM and its association with CPM efficacy. This study found reduced vagal activity during the pressure threshold test with conditioning painful stimulus (∼20 seconds), which was not associated with the CPM magnitude.^35^ That study used heart rate variability (HRV) as a noninvasive index of vagal function during pain.^34^ However, recent evidence highlights concerns about the accuracy and reliability of ultra-short HRV recordings (ie, recordings shorter than 5 minutes),^11,69^ suggesting that recording duration should exceed 40 seconds.^30^ Therefore, vagal reactivity should be tested during sustained painful stimulation.

This study aimed to examine the vagal–CPM association in healthy females, as part of a broader investigation focused on the female population. We hypothesized that resting vagal tone would interact with menstrual cycle phase to predict CPM magnitude and that psychological factors would mediate this association. In addition, we hypothesized that greater vagal reactivity during CPM would be associated with more efficient CPM.

## 2. Methods

The study was approved by the University of Haifa Ethics Committee (approval number 438/21).

### 2.1. Participants

Healthy females between 18 and 40 years old were recruited over 1 year (December 2023–2024), via the university research participation system. Exclusion criteria included (1) attention deficit hyperactivity disorder diagnosis; (2) chronic and acute pain; (3) current pregnancy; (4) using hormonal contraceptives or a hormone-coated implantable device; (5) irregular menstrual cycles, or cycle lengths shorter than 21 days or longer than 35 days; and (6) regular use of analgesics and psychiatric medications in the last 3 months. These criteria were assessed using a structured self-report medical history questionnaire. Participants were instructed to avoid caffeine for 3 hours and analgesics for 24 hours before testing. All provided informed consent and received course credit or financial compensation.

### 2.2. Autonomic nervous system assessment

#### 2.2.1. Heart rate variability

Electrocardiography (ECG) was recorded using a BIOPAC MP150 system (BIOPAC Systems Inc, Goleta, CA) at 1000 Hz, with electrodes placed in a three-lead chest-mounted configuration (lead II). During the recordings, participants were asked to sit quietly and breathe normally. A 5-minute baseline ECG was collected at the start of the experiment (rest HRV). In addition, ECG was recorded 60 seconds before (baseline), during (reactivity), and after (recovery) the pain test stimulus, both alone and with conditioning stimulus. Signals were preprocessed using Neurokit2 (v0.2.7)^42^ with a 5-Hz low-pass and 50-Hz notch filter. According to published guidelines,^29,37^ QRS complexes (QRS; the waveform representing ventricular depolarization) were detected based on the steepness of the absolute gradient, with R-peaks detected as local maxima of each QRS. Artifacts were corrected based on beat classification, and cubic trends were removed.

R–R intervals were used for calculating HRV time and frequency measures, selected according to established guidelines.^18,45,52^ In the time domain, the root mean square of successive differences (RMSSD) between adjacent R–R intervals was calculated as the primary metric indicative of vagal tone.^37^ In the frequency domain, high-frequency power (HF; 0.15–0.4 Hz, indicative of vagal activity), low-frequency power (LF; 0.04–0.15 Hz, reflective combination of sympathetic and vagal activity), and the LF/HF ratio (LF/HF, an index of sympatho-vagal balance) were assessed. Heart rate variability measures were log-transformed due to non-normal distributions, following recommendations.^37,68^ Mean heart rate (HR) was also calculated and used in its raw form, as it was normally distributed.

Electrocardiography at 5-minute rest was analyzed at the time and frequency domains of HRV (ie, *rest HRV*). Electrocardiography at a 1-minute baseline, reactivity, and recovery were analyzed only at the time domain of HRV (ie, RMSSD), as 1-minute recording is not considered appropriate for obtaining reliable frequency domain measures.^11,68^ Further, HRV and HR values that were 3 z-scores above or below the participants' mean in each measurement time were removed from the analysis (<1% of all the measurements).

### 2.3. Quantitative sensory testing

#### 2.3.1. Determination of pain-60 temperature

The temperature corresponding to participants' pain-60 (ie, heat temperature rated as 60 on a 0–100 numerical pain scale [NPS], “0” = no pain, “100” = worst imaginable pain) was determined. Participants received varied 8-second heat stimuli using a 30 × 30 cm Peltier thermode (TSA-2001, Medoc, Ramat Yishai, Israel) applied to the volar forearm of the dominant hand (2°C/second ramp from 32°C baseline) and were asked to rate each on the NPS. The thermode was repositioned after each stimulus to avoid sensitization. This process continued until the same temperature elicited a rating of ∼60 in 3 separate trials. This temperature was defined as pain-60 and was used for the tonic heat stimulation.^22^

#### 2.3.2. Tonic heat pain

The thermode was applied to the volar forearm of the dominant hand. The temperature increased from 32°C to the participant's pain-60 at 2°C/second and was held constant for 60 seconds. Participants rated the pain on the NPS at target temperature onset and every 10 seconds thereafter. The average of these 7 ratings served as the tonic heat pain (THP) score and was used as the test stimulus in the CPM paradigm.

#### 2.3.3. Conditioned pain modulation

Conditioned pain modulation was assessed using a standardized protocol.^23,31,55,83^ Participants immersed their nondominant hand in a hot water bath (Heto Cooling Bath CBN 8–30, Denmark), which served as the *conditioning stimulus*. Water temperature ranged between 44 and 47°C (mean: 45.24°C [*0.74*]), adjusted to mild–moderate pain range (ie, NPS 20–80), following standard recommendations.^83^ Hot water conditioning was selected over cold water to avoid sympathetic activation,^72^ potentially confounding the assessment of pain-related autonomic responses. After 20 seconds, participants were asked to rate pain intensity on the NPS and turn their attention to the dominant hand while keeping the nondominant hand immersed. Then, the THP test stimulus was applied for 60 seconds, during which participants rated the pain intensity every 10 seconds. After THP termination, participants rated the hot water pain again before removing their hand. *CPM magnitude* was calculated by subtracting the mean pain ratings of the THP stimulus given alone from the mean THP ratings during the conditioning stimulus, such that a more negative CPM magnitude score represented better pain inhibition.^83^

### 2.4. Self-reported trait pain-related psychological questionnaires

To assess psychological factors related to pain, participants completed the Hebrew versions of 3 validated questionnaires. *The Pain Catastrophizing Scale (PCS)*^71^ consists of 13 items assessing rumination, magnification, and helplessness related to past pain experiences, rated on a 5-point scale; internal consistency in our sample was high (α = 0.93). *The Fear of Pain Questionnaire (FPQ-9)*^44^ measures fear of minor, severe, and medical pain on a 5-point scale, with good reliability in our sample (α = 0.85). Finally, *the Depression, Anxiety, and Stress Scale (DASS-21)*^40^ evaluates negative emotional states over the past week using 21 items rated on a 4-point scale; reliability was high in our sample (α = 0.91).

### 2.5. Experimental procedure

Upon arrival, participants were informed about the experiment, and the inclusion criteria were verified. Then, they were connected to the ECG electrodes and were instructed to sit quietly and rest for 5 minutes of continuous heartbeat recording.

Next, participants completed demographic questions (eg, sex, age, ethnicity, body mass index), and the first day of menstrual bleeding to determine the menstrual cycle phase (using the counting method, based on Ref. 66), and self-reported questionnaires (ie, PCS, FPQ-9, and DASS-21). Then, the quantitative sensory testing assessment was conducted, starting with familiarization, pain-60 determination, and 60-second forearm THP testing, alone and with the conditioning stimulus. During the THP, both alone and during conditioning, pain ratings were provided every 10 seconds using the NPS. Electrocardiography was recorded 60 seconds before (baseline), during (reactivity), and after (recovery) the THP alone and during conditioning.

### 2.6. Statistical analysis

Statistical analyses were performed using R Software (v4.1.2; R Core Team 2021), with the lmer^6^ and the interaction^39^ packages. All analyses were corrected for multiple comparisons using Bonferroni correction, with significance set at *P* < 0.05 for each hypothesis.

A priori power analyses were conducted using G*Power 3.1.9.7^17^ to determine the sample size required to test our hypotheses. For the repeated-measures ANOVA (6 measurements: 2 pain conditions × 3 HRV phases, with 3 covariates: pain-60 temperature, conditioning pain rating, and menstrual cycle phase), a minimum of 55 participants was required to achieve 80% power at α = 0.05. For the multiple regression predicting CPM magnitude from 5 predictors (3 autonomic variables, menstrual cycle phase, and pain-60 temperature), 65 participants were needed. To account for dropout and missing data, the target sample size was increased by 15% to 75 participants.

Normality was assessed using histograms and the Shapiro–Wilk test. Due to high correlations with RMSSD, HF was excluded to avoid multicollinearity. No multicollinearity was found among psychological variables.

To evaluate the effect of the CPM paradigm conditioning, a 2-way repeated-measures ANOVA was conducted on pain ratings over time (0–60 seconds) across 2 conditions (THP alone vs with conditioning). Conditioned pain modulation efficiency was assessed via the interaction effect, reflecting greater pain reduction under the CPM condition.

To test whether vagal tone interacts with menstrual cycle phase in predicting pain inhibition, we conducted 2 multiple regressions: one for THP ratings alone, and another for CPM magnitude. Predictors included baseline autonomic measures at 5-minute rest (LogRMSSD, LogLF, LogLF/HF), pain-60 temperature, menstrual cycle phase (as a categorical variable, based on its common division into the follicular phase, which begins with the onset of menstruation and continues until the end of ovulation, typically lasts around 15 days, and the luteal phase, which starts after ovulation and ends just before the next menstruation),^47^ and the interaction between menstrual cycle phase and LogRMSSD. Post-hoc analysis was then used to identify in which menstrual cycle phases the LogRMSSD–CPM relationship was significant. Although our focus was on vagal tone, sympathetic measures were included to control for their effects. Further, the actual pain-60 temperature was included as it was previously shown to influence CPM magnitude beyond subjective ratings.^61,83^ All assumptions for moderation analysis (linearity, normality, homoscedasticity, no multicollinearity, and independent errors) were met, and predictors were mean-centered.

To test whether psychological factors (distress, fear of pain, pain catastrophizing) mediate the vagal tone–CPM association, correlation analysis was first conducted, followed by mediation analysis if both CPM magnitude and vagal tone were significantly related to these factors.

Last, to test our hypothesis regarding vagal reactivity during THP alone and under conditioning, a two-way repeated measures ANOVA was conducted on logRMSSD across 3 stages (baseline, reactivity, recovery) in both conditions. Pain-60 temperature, mean conditioning pain ratings (average of water pain ratings), and menstrual cycle phase (as a categorical variable) were included as covariates to control for their influence.^45,80^ The same model was used to assess heart rate changes across stages. Next, regression analyses tested whether vagal reactivity (logRMSSD during pain) and RMSSD %change from baseline (calculated as: [RMSSD during reactivity-RMSSD at baseline]/baseline RMSSD) predicted THP ratings and CPM magnitude. Menstrual cycle phase (categorical) was included to examine its effect on these associations.

## 3. Results

### 3.1. Participants

Seventy-five healthy females participated in the study, which is an independent part of a larger study examining the effect of cognitive-emotional trainings on pain. Due to technical issues, 5 participants did not complete the study, and data from an additional 3 were incomplete and excluded. Thus, the final sample size included 67 participants (mean age 23.21 ± 3; range 18–33 years).

#### 3.1.1. Demographic and autonomic characteristics

Demographic information and psychological characteristics of the participants are presented in Table 1. Descriptive statistics of HRV measures taken at 5-minute rest are depicted in Table 2. Due to a technical issue, rest ECG recording of 1 participant was not obtained.

**Table 1:** Participant demographics and psychological characteristics.

	(N = 67)
Age (y)	
Mean (SD)	23.16 (3.0)
Range (Min–Max)	18–33
Body mass index (BMI)	
Mean (SD)	22.18 (3.1)
Range (Min–Max)	17–32
Dominant hand	
Right hand (%)	60 (89.5%)
Left hand (%)	7 (10.5%)
Ethnicity	
Jews (%)	32 (52.2%)
Arabs (%)	35 (47.8%)
Menstrual cycle phase	
Follicular (%)	37 (55.2%)
Luteal (%)	30 (44.7%)
Pain catastrophizing scale (PCS)	
Mean (SD)	16.96 (10.6)
Range (Min–Max)	0–42
Fear of pain questionnaire (FPQ-9)	
Mean (SD)	25.48 (6.8)
Range (Min–Max)	9–40
Depression, anxiety, and stress scales - short form (DASS-21)	
Mean (SD)	11.78 (9.3)
Range (Min–Max)	0–46

Mean scores are presented with standard deviation in parentheses.

**Table 2:** Descriptive statistics of all heart rate variability measures at baseline 5-minute rest.

HRV measures at 5-min rest	(N = 66)
RMSSD (ms^2^)	
Mean (SD)	37.60 (21.32)
Range (Min–Max)	8–109
LF (ms^2^)	
Mean (SD)	1018.48 (1128.2)
Range (Min–Max)	75–6902
LF/HF (ms^2^)	
Mean (SD)	1.89 (1.4)
Range (Min–Max)	0.2–7.5
HR (bpm)	
Mean (SD)	82.83 (11.6)
Range (Min–Max)	62–113

HR, heart rate (bpm); HRV, heart rate variability; LF, low-frequency power (ms^2^); LF/HF, The ratio of low-frequency power to high-frequency power; RMSSD, root mean square of successive difference (ms^2^).

#### 3.1.2. Pain ratings during tonic heat pain

The pain-60 temperatures used for the THP test ranged between 41 and 48°C with a mean temperature of 45.07°C (*1.88*). The mean pain intensity ratings during THP was 31.9 (*17*) NPS. Mean pain rating at THP onset was 59.15 (*19.1*) NPS, indicating a successful determination of participants' pain-60.

#### 3.1.3. Pain ratings during tonic heat pain with a conditioning stimulus

**Figure 1.**
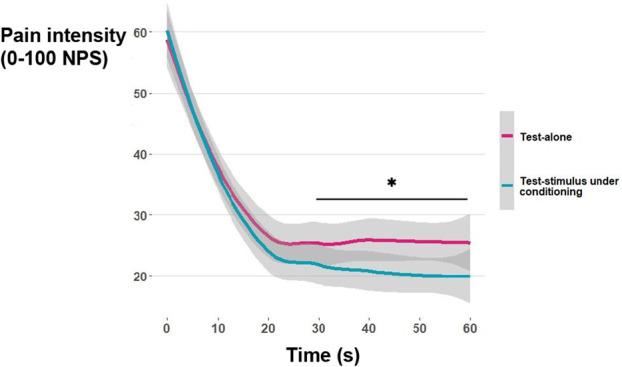
Pain ratings during 1-minute THP given alone and under conditioning. Pain intensity ratings were rated verbally every 10 seconds. Although there was no difference between pain ratings at the first 20 seconds (all *Ps* > 0.05), from 30 seconds on, the ratings of THP with conditioning stimulus were lower compared to THP given alone. For ease of viewing, the *y*-axis range is presented between 20 and 60 NPS, although participants rated their pain on a 0 to 100 NPS scale. *P < 0.05. CPM, conditioned pain modulation; NPS, numerical pain scale; THP, tonic heat pain.

Table 3 describes the THP ratings alone and with a conditioning stimulus. The ANOVA analysis results indicate that pain ratings where higher during THP alone compared to THP with conditioning (F_(1,66)_ = 5.39, *P* = 0.024, η^2^ = 0.075), suggesting a significant pain inhibition. Further, pain ratings decreased over time (F_(2,66)_ = 67.75, *P* < 0.001, η^2^ = 0.51), with a significant interaction showing that the change over time differed between conditions (F_(6,61)_ = 3.53, *P* = 0.002, η^2^ = 0.051). Post-hoc analysis with Bonferroni correction indicated that there was no difference in pain ratings between THP alone and with conditioning during the first 20 seconds (*P*s > 0.05); however, starting from 30 seconds, pain ratings during conditioning were reduced compared to THP alone (*P* < 0.023; Fig. 1).

**Table 3:** Mean and SD of pain intensity ratings on the 0–100 numerical pain scale during tonic heat pain given alone and with conditioning stimulus (under the conditioned pain modulation paradigm).

Condition	At stimulus onset	After 10 s	After 20 s	After 30 s	After 40 s	After 50 s	After 60 s	Mean rating
THP given alone	59.15 (19.1)	38.55 (21)	28.01 (21.7)	26.84 (23.6)	27.73 (26.6)	26.06 (26.7)	27.52 (26.9)	31.9 (17)
THP with a conditioning stimulus (CPM paradigm)	62.09 (18.5)	34.13 (22.8)	27.07 (23.1)	22.75 (23.3)	21.72 (23.2)	21.94 (24.1)	20.93 (24.7)	28.29 (18.1)
	*P* = 0.204	*P* = 0.142	*P* = 0.688	*P* = 0.024	*P* = 0.003	*P* = 0.023	*P* < 0.001	*P* = 0.043

*P* values represent the difference between the pain rating of THP given alone and with a conditioning stimulus in each time point. *P* values are presented with Bonferroni correction for multiple comparisons.

CPM, conditioned pain modulation paradigm; THP, tonic heat pain.

### 3.2. Predicting tonic heat pain ratings and conditioned pain modulation magnitude based on baseline characteristics

The results for the regression model showed that among all variables (Table 4), only pain-60 temperature (β = 0.31, *P* = 0.019) predicted higher mean THP alone ratings (F_(6,57)_ = 2.41, *P* = 0.038, adjusted R^2^ = 0.12).

**Table 4:** Results of the multiple regression analyses for predicting pain ratings of tonic heat pain administered alone and conditioned pain modulation magnitude based on baseline autonomic variables at rest.

Predicted Condition	Predictors	b (Unstandardized)	SD	β (Standardized)	95% CI	*t*	*P*
Pain ratings of THP given alone	LogRMSSD	31.06	19.36	0.37	−7.72 to 69.85	1.60	*0.114*
	LogLF	−5.51	8.36	−0.13	−22.17 to 11.15	−0.66	*0.510*
	Log LF/HF	3.83	8.09	0.07	−12.38 to 20.05	0.47	*0.638*
	Menstrual cycle phase	4.80	4.28	0.14	−3.78 to 13.38	1.12	*0.267*
	LogRMSSD × menstrual cycle phase	0.49	23.10	0.00	−45.80 to 46.77	0.02	*0.983*
	Pain-60 temperature	2.86	1.18	0.31	0.49 to 5.23	2.42	***0.019***
CPM magnitude	LogRMSSD	−46.52	17.58	−1.05	−81.72 to −11.32	−2.65	***0.010***
	LogLF	18.50	9.49	0.68	−0.51 to 37.50	1.95	*0.056*
	Log LFHF	−6.42	8.24	−0.19	−22.92 to 10.08	−0.78	*0.439*
	Menstrual cycle phase	−1.88	2.58	−0.17	−7.04 to 3.28	−0.73	0.469
	LogRMSSD × menstrual cycle phase	27.67	11.51	0.62	4.62 to 50.72	2.40	***0.020***
	Pain-60 temperature	0.98	0.69	0.17	−0.40 to 2.36	1.42	0.161

*P*-values are presented in italics, and statistically significant *P*-values are highlighted in bold.

CI, Confidence interval; CPM, conditioned pain modulation; LF, low-frequency power (ms^2^); LF/HF, The ratio of low-frequency power to high-frequency power; Menstrual cycle phase, as a categorical variable, based on its common division into the follicular phase and the luteal phase; Pain-60 temperature, the temperature participants rated as 60 on 0 to 100 NPS; RMSSD, root mean square of successive difference (ms^2^); THP, tonic heat pain.

**Figure 2.**
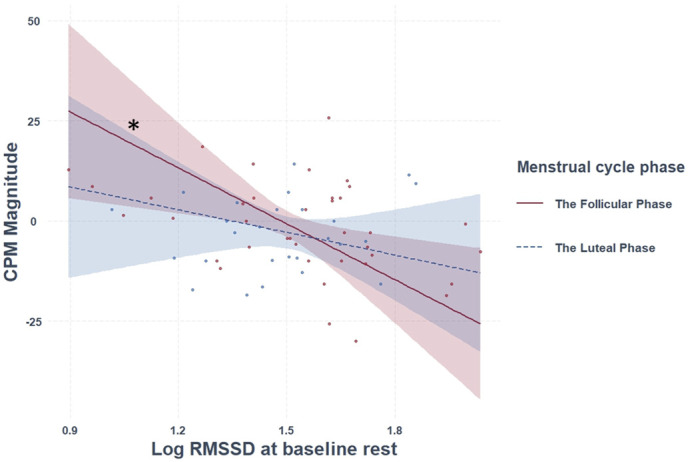
The relationship between logRMSSD at 5-minute baseline rest and CPM magnitude, as a function of the menstrual cycle phase. The relationship between logRMSSD at rest and CPM magnitude was significant only for participants at the follicular phase. *P < 0.05. CPM, conditioned pain modulation; RMSSD, root mean square of successive differences.

On the contrary, CPM magnitude (F_(6,57)_ = 3.146, *P* = 0.010, adjusted R^2^ = 0.170) was predicted by a main effect of logRMSSD at rest (β = −0.79, *P* = 0.010), and by an interaction between the menstrual cycle phase and logRMSSD (β = 0.31, *P* = 0.020). Post-hoc analysis revealed that higher logRMSSD at rest predicted better CPM magnitude for participants at the follicular phase (β = −1.05, *P* = 0.010; Fig. 2), but not in the luteal phase (β = −0.42, *P* = 0.31).

### 3.3. Psychological factors as mediators of the association between resting vagal tone and conditioned pain modulation

There were no associations between psychological measures (DASS-21, PCS, FPQ9), CPM magnitude, and resting logRMSSD (Table 5). As such, mediation analysis was not pursued, given the absence of the required correlations necessary for mediation.

**Table 5:** Correlations between psychological variables, conditioned pain modulation magnitude, and logRMSSD.

	Depression (DASS)	PCS	FPQ-9	CPM magnitude	LogRMSSD
Depression (DASS)	—	0.38**	0.26*	0.02	−0.07
PCS	0.38**	—	0.4***	0.03	−0.09
FPQ-9	0.26*	0.4***	—	−0.01	0.08
CPM magnitude	0.02	0.03	−0.01	—	−0.16
LogRMSSD	−0.07	−0.09	0.08	−0.16	—

*P < 0.05; **P < 0.01; ***P < 0.001.

CPM, conditioned pain modulation, ie, tonic heat pain test stimulus with hot water pain conditioning; DASS, the short depression, anxiety and stress scale; FPQ-9, fear of pain; PCS, pain catastrophizing scale.

### 3.4. Vagal and heart rate reactivity during tonic heat pain alone and with conditioning stimulus

Descriptive statistics for RMSSD and HR at a 1-minute baseline, reactivity, and recovery are presented in Table 6. LogRMSSD changed between the stages (F_(2,65)_ = 13.74, *P* < 0.001, η^2^ = 0.172), and pain conditions (F_(1,66)_ = 13.13, *P* < 0.001, η^2^ = 0.166), a was overall lower during THP with conditioning. In addition, an interaction between phase and condition was found (F_(2,132)_ = 3.8, *P* = 0.025, η^2^ = 0.055). Post-hoc analysis revealed that logRMSSD decreased from baseline to reactivity in both conditions (THP alone: Δ = −0.05, 95% CI [−0.08 to 0.04], *P* = 0.009; CPM paradigm: Δ = −0.10, 95% CI [−0.14 to 0.06], *P* = 0.009). During reactivity, logRMSSD was lower during THP with conditioning compared to THP alone (Δ = −0.06, 95% CI [−0.10 to 0.03], *P* = 0.009) and remained lower during recovery (Δ = −0.07, 95% CI [−0.08 to 0.02], *P* = 0.009; Table 6).

**Table 6:** Descriptive statistics of root mean square of successive differences and heart rate at 1-minute baseline, reactivity, and recovery for the tonic heat pain given alone and with conditioning.

Condition	Baseline		Reactivity				Recovery	
	RMSSD	HR	RMSSD	RMSSD %change from baseline	HR	HR % change from baseline	RMSSD	HR
THP given alone								
Mean (SD)	45.48 (23.3)	78.04 (10.6)	39.36 (18.2)	−7.4% (23.3)	78.35 (10.9)	2% (5.0)	44.52 (21.9)	79.36 (10.4)
Range	10.9–115.9	58.62–99.9	13.2–94.2	−51% to +66%	58.1–103.7	−8% to +15%	9.5–113.9	57.9–104.2
THP with a conditioning stimulus (CPM paradigm)								
Mean (SD)	45.79 (25.7)	78.11 (10.9)	34.84 (18)	−18.9% (25.8)	83.4 (10.7)	7.4% (6.1)	37.81 (18.2)	82.37 (10.2)
Range	9.8–118.6	57.8–101.4	8.0–103.5	−65% to +77%	59.4–110.9	−3% to +19%	10.8–100.7	61.5–111.6

For ease of understanding, RMSDD values are presented in absolute values. However, due to the no-linearity of this measurement, the analyses were conducted based on RMSSD log transformation.

CPM, conditioned pain modulation; HR, heart rate; RMSSD, root mean square of successive difference (ms^2^); RMSSD % change from baseline, the difference between RMSSD during reactivity and RMSSD at baseline, divided by the baseline RMSSD; THP, tonic heat pain.

Nevertheless, when the same analysis was conducted, controlling for pain-60 temperature, conditioning stimulus pain ratings, and menstrual cycle phase, these effects were not significant anymore (all *P*s > 0.3).

Heart rate also changed between the stages (F_(2,132)_ = 29.7, *P* < 0.001, η^2^ = 0.311), and conditions (F_(1,66)_ = 82.5, *P* < 0.001, η^2^ = 0.556), and was overall elevated during the CPM paradigm. In addition, an interaction between stage and condition was found (F_(2,132)_ = 47.82, *P* < 0.001, η^2^ = 0.42). Post-hoc analysis revealed that under THP alone, HR was elevated during recovery compared to baseline (Δ = 1.32, 95% CI [0.47–2.1], *P* = 0.018). During conditioning, HR increased from baseline to reactivity (Δ = 5.28, 95% CI [4.23–6.48], *P* < 0.001) and remained elevated at recovery (Δ = 4.25, 95% CI [3.2–5.3], *P* = 0.009). In addition, HR reactivity was significantly higher during conditioning compared to THP alone (Δ = 5.04, 95% CI [4.17–5.89], *P* = 0.009), and this difference persisted during recovery (Δ = 3.00, 95% CI [2.06–3.94], *P* = 0.009; Table 6).

When the same analysis was conducted while controlling for pain-60 temperature, conditioning stimulus pain ratings, and menstrual cycle phase, HR changes were no longer influenced by the conditions or stage (all *P*s > 0.3). However, conditioning stimulus pain ratings significantly influenced HR, with higher pain ratings linked to increased HR during THP with conditioning (F_(1,63)_ = 5.95, *P* = 0.018, η^2^ = 0.086).

### 3.5. The association between vagal reactivity, tonic heat pain pain ratings, and the conditioned pain modulation magnitude

Higher logRMSSD during THP alone correlated with higher pain ratings (r = 0.29, *P* = 0.023). This association was not affected by menstrual cycle phase (*P* = 0.98). RMSSD %change was not related to THP ratings (*P* = 0.066) and showed no interaction with the menstrual cycle (*P* = 0.083).

In contrast, higher logRMSSD during THP with conditioning was associated with greater CPM magnitude (r = −0.36 *P* = 0.004). This association was not affected by the menstrual cycle phase (*P* = 0.57). RMSSD %change was unrelated to CPM magnitude (*P* = 0.20) and showed no interaction with the menstrual cycle (*P* = 0.081).

## 4. Discussion

In this study, we investigated the vagal–CPM association in a cohort of healthy females. Higher CPM magnitude was predicted by an interaction between the vagal tone and the menstrual cycle phase, with the vagal–CPM association occurring only in the follicular phase. Contrary to our assumption, psychological factors did not mediate the vagal–CPM association.

We further hypothesized that vagal reactivity during CPM would be linked to more efficient pain inhibition. Although group-level analyses showed reduced vagal activity during THP alone and during THP with conditioning stimulus, this effect was not significant after controlling for covariates (conditioning pain ratings, test stimulus temperature, and menstrual cycle phase). Still, vagal activity during CPM varied widely and was positively correlated with CPM magnitude.

### 4.1. Test characteristics but not autonomic activity predict tonic heat pain ratings

When THP was administered alone, pain ratings were predicted solely by stimulus temperature, a well-established relationship.^80^ However, baseline HRV did not predict pain ratings, diverging from previous findings linking higher resting LF-HRV or HF-HRV with lower pain sensitivity.^2,76^ Recent meta-analyses emphasize the complexity of this relationship, noting that factors such as expectations, stimulation parameters, and measurement methods may influence autonomic-pain associations.^18,19,34^ Future research should systematically evaluate the contribution of these variables to clarify the link between resting autonomic activity and pain perception.

### 4.2. The interactive effect of the menstrual cycle phase on the vagal–conditioned pain modulation association

The role of autonomic activity and, specifically, the vagal tone in CPM efficiency remains unclear. Although some studies report that higher vagal tone predicts greater CPM magnitude,^78^ this effect appears limited to males,^49^ individuals with low-to-moderate physical activity,^46^ or highly anxious females.^51^ Other studies report no association at all.^41,54^ Similarly, vagus nerve stimulation yields inconsistent effects on CPM; although two studies reported enhanced CPM,^58,85^ one found no effect.^1^ This inconsistency suggests that other factors may interact with the vagal–CPM association. In our study, higher vagal tone at rest predicted better CPM efficiency only among females in the follicular phase.

The menstrual cycle's role in the vagal–CPM association may reflect fluctuations in vagal tone across cycle phases. A recent meta-analysis found that vagal tone decreases from the follicular to the luteal phase,^65^ which may be explained by fluctuations in ovarian hormones—namely, estrogen and progesterone—across the menstrual cycle. Estrogen peaks during mid-follicular and drops postovulation, whereas progesterone rises after ovulation and declines premenstrually.^4^ The abrupt premenstrual decline in progesterone and estrogen levels may reduce inhibitory control over the central nucleus of the amygdala due to GABAergic withdrawal.^4,10^ This disinhibition can, in turn, activate sympathoexcitatory neurons in the rostral ventrolateral medulla, resulting in heightened sympathetic nervous system activity.^67,74^ Simultaneously, vagal activity may decrease due to the inhibition of the tonically active neurons in the vagal motor nucleus.^67,74^ Together, these mechanisms are likely key contributors to the reduction in vagally mediated HRV during later menstrual phases.^65^

The complex effects of ovarian hormones on vagal activity may explain inconsistent findings regarding associations between menstrual cycle phases and CPM efficacy. Although some studies report greater pain inhibition during ovulation compared to the follicular and luteal phases,^26,63,75^ others find no association.^5,79,81^ These discrepancies may reflect individual differences in hormone levels and cycle characteristics, which influence hormonally mediated vagal changes.^24,38^ For example, a recent study found that, beyond group-level reductions in HF-HRV during the luteal phase, higher-than-usual progesterone levels within an individual predicted lower-than-usual HF-HRV.^64^ This finding highlights the importance of considering both between- and within-subject hormonal variability when examining the menstrual cycle's impact on autonomic regulation and pain modulation.

### 4.3. Limited role of psychological factors in the vagal–conditioned pain modulation association

Unlike the menstrual cycle involvement in the vagal–CPM association, we did not find a correlation between distress, fear of pain, and pain catastrophizing with CPM, nor an interaction effect with vagal tone. Although previous studies have linked these variables to CPM efficiency,^14,51^ their combined contribution was minimal, explaining only 3% of the variance in CPM magnitude,^21^ with effects largely limited to modality-specific responses.^50^ Thus, in healthy individuals, psychological influences on CPM may be limited or context-dependent.

### 4.4. Greater vagal activity during the test stimulus with conditioning is associated with more efficient pain inhibition

Autonomic–baroreflex interactions are key to maintaining homeostasis during pain.^70^ In this study, we evaluated RMSSD-HRV as a measure of the efferent pathways of the vagal baroreflex, which regulates HR and inhibits pain.^12,69^ To our knowledge, this is the first study investigating vagal reactivity to continuous pain during CPM, mandatory for HRV evaluation.^11^ Although RMSSD decreased from baseline during both THP alone and with conditioning, these changes were not significant after controlling for conditioning stimulus ratings, test stimulus temperature, and menstrual cycle phase, aligning with studies showing that CPM magnitude^28^ and vagal response^34^ are affected by testing characteristics.

Notably, individuals with greater vagal activation (or less vagal withdrawal) during CPM showed more effective pain inhibition. According to the neurovisceral integration model^73^ and polyvagal theory,^60^ tonic (resting) and phasic (reactive) vagal control are associated differently with regulation and adaptability functions. Higher tonic vagal tone is commonly linked to enhanced executive functioning, emotional regulation, and stress resilience.^36^ In contrast, adaptive phasic vagal responses are context-dependent: greater vagal withdrawal may be more adaptive in high-demand, metabolically taxing situations, whereas vagal maintenance, or reduced withdrawal, may be more beneficial during tasks requiring sustained inhibition, such as pain processing.^59,65^ Accordingly, women who maintained or increased vagal reactivity during dual pain exhibited stronger pain inhibition, possibly reflecting more effective engagement of baroreflex-mediated endogenous pain inhibition,^16^ independent of menstrual phase. These findings suggest a complex interplay between hormonal states and vagal modulation in shaping pain regulatory capacity, aligning with early evidence from animal models.^32,33,82^ Further research is needed to clarify hormone-specific patterns of vagal dynamics in pain regulation.

### 4.5. Limitations

This study examined the vagal–CPM association exclusively in healthy young females, limiting generalizability to males and older females, given hormonal changes postmenopause^47^ and age-related HRV decline.^43^ Furthermore, menstrual cycle phase was determined using the counting method, a common practice in studies on menstrual effects, but not entirely accurate due to inter- and intraindividual variability in cycle length.^66^ Consequently, the interpretation of menstrual effects should be made with caution, particularly given the cross-sectional nature of our design. Future studies should consider employing hormonal verification and a within-subject longitudinal study design to more precisely assess the influence of menstrual phase on the vagal–CPM relationship.

## 5. Conclusions

Our findings give initial evidence indicating that the vagal–CPM association emerges only in the early menstrual phase, highlighting the influence of hormonal fluctuations on vagal pain modulation. Significant interindividual variability in vagal activity during CPM was also observed, relevant given the documented contribution of both impaired autonomic regulation and dysfunctional endogenous pain inhibition to chronic pain. To our knowledge, this is the first study exploring whether menstrual cycle phase modulates the vagal–CPM relationship, introducing a previously unexplored psychophysiological interaction. Although the cross-sectional design limits causal interpretation and precise cycle phase verification, the results offer valuable foundations for future longitudinal research aimed at clarifying the mechanisms linking vagal activity, menstrual cycle, and pain regulation in women.

## Disclosures

This work was supported by the Artificial Intelligence Research Center (HiAI) at the University of Haifa, awarded to all authors. The authors declare no conflicts of interest. Data from the current experiment will be made available upon request.

## References

[1] Alt LK Wach K Liebler EJ Straube A Ruscheweyh R. A randomized Sham-controlled cross-over study on the short-term effect of non-invasive cervical vagus nerve stimulation on spinal and supraspinal nociception in healthy subjects. Headache 2020;60:1616–31. 32592516 10.1111/head.13891

[2] Appelhans BM Luecken LJ. Heart rate variability and pain: associations of two interrelated homeostatic processes. Biol Psychol 2008;77:174–82. 18023960 10.1016/j.biopsycho.2007.10.004

[3] Bai X Li J Zhou L Li X. Influence of the menstrual cycle on nonlinear properties of heart rate variability in young women. Am J Physiol Heart Circulat Physiol 2009;297:H765–74. 10.1152/ajpheart.01283.2008 19465541

[4] Barbas H Saha S Rempel-Clower N Ghashghaei T. Serial pathways from primate prefrontal cortex to autonomic areas may influence emotional expression. BMC Neurosci 2003;4:25. 14536022 10.1186/1471-2202-4-25 PMC270042

[5] Bartley EJ Rhudy JL. Endogenous inhibition of the nociceptive flexion reflex (NFR) and pain ratings during the menstrual cycle in healthy women. Ann Behav Med 2012;43:343–51. 22289982 10.1007/s12160-012-9345-x

[6] Bates D Mächler M Bolker B Walker S. Fitting linear mixed-effects models using lme4. J Stat Softw. 2015;67. doi:10.18637/jss.v067.i01.

[7] Benarroch EE. Descending monoaminergic pain modulation. Neurology 2008;71:217–21. 18625968 10.1212/01.wnl.0000318225.51122.63

[8] Berthoud H-R Neuhuber WL. Functional and chemical anatomy of the afferent vagal system. Auton Neurosci 2000;85:1–17. 11189015 10.1016/S1566-0702(00)00215-0

[9] Bonaz B Sinniger V Pellissier S. Vagal tone: effects on sensitivity, motility, and inflammation. Neurogastroenterol Motil 2016;28:455–62. 27010234 10.1111/nmo.12817

[10] Brunton PJ Donadio MV Yao ST Greenwood M Seckl JR Murphy D Russell JA. 5α-Reduced neurosteroids sex-dependently reverse central prenatal programming of neuroendocrine stress responses in rats. J Neurosci 2015;35:666–77. 25589761 10.1523/JNEUROSCI.5104-13.2015 PMC4293416

[11] Burma JS Graver S Miutz LN Macaulay A Copeland PV Smirl JD. The validity and reliability of ultra-short-term heart rate variability parameters and the influence of physiological covariates. J Appl Physiol 2021;130:1848–67. 33856258 10.1152/japplphysiol.00955.2020

[12] Carrasco-Sosa S Gaitán-González MJ González-Camarena R Yáñez-Suárez O. Baroreflex sensitivity assessment and heart rate variability: relation to maneuver and technique. Eur J Appl Physiol 2005;95:265–75. 16086148 10.1007/s00421-005-0001-z

[13] Chakravarthy K Chaudhry H Williams K Christo PJ. Review of the uses of vagal nerve stimulation in chronic pain management. Curr Pain Headache Rep 2015;19:54. 26493698 10.1007/s11916-015-0528-6

[14] Christensen KS O'Sullivan K Palsson TS. Conditioned pain modulation efficiency is associated with pain catastrophizing in patients with chronic low back pain. Clin J Pain 2020;36:825–32. 32815869 10.1097/AJP.0000000000000878

[15] De Couck M Nijs J Gidron Y. You may need a nerve to treat pain: the neurobiological rationale for vagal nerve activation in pain management. Clin J Pain 2014;30:1099–105. 24451632 10.1097/AJP.0000000000000071

[16] Duschek S Werner NS Reyes del Paso GA. The behavioral impact of baroreflex function: a review. Psychophysiology 2013;50:1183–93. 24033333 10.1111/psyp.12136

[17] Faul F Erdfelder E Lang A-G Buchner A. G* Power 3: a flexible statistical power analysis program for the social, behavioral, and biomedical sciences. Behav Res Methods 2007;39:175–91. 17695343 10.3758/bf03193146

[18] Firouzian S Osborne NR Cheng JC Kim JA Bosma RL Hemington KS Rogachov A Davis KD. Individual variability and sex differences in conditioned pain modulation and the impact of resilience, and conditioning stimulus pain unpleasantness and salience. PAIN 2020;161:1847–60. 32701844 10.1097/j.pain.0000000000001863

[19] Forte G Troisi G Pazzaglia M Pascalis VD Casagrande M. Heart rate variability and pain: a systematic review. Brain Sci 2022;12:153. 35203917 10.3390/brainsci12020153 PMC8870705

[20] Frangos E Richards EA Bushnell MC. Do the psychological effects of vagus nerve stimulation partially mediate vagal pain modulation? Neurobiol Pain 2017;1:37–45. 29057372 10.1016/j.ynpai.2017.03.002 PMC5648334

[21] Graeff P Itter A Wach K Ruscheweyh R. Inter-individual differences explain more variance in conditioned pain modulation than age, sex and conditioning stimulus intensity combined. Brain Sci 2021;11:1186. 34573207 10.3390/brainsci11091186 PMC8468738

[22] Granot M Weissman-Fogel I Crispel Y Pud D Granovsky Y Sprecher E Yarnitsky D. Determinants of endogenous analgesia magnitude in a diffuse noxious inhibitory control (DNIC) paradigm: do conditioning stimulus painfulness, gender and personality variables matter? PAIN 2008;136:142–9. 17720319 10.1016/j.pain.2007.06.029

[23] Granovsky Y Miller-Barmak A Goldstein O Sprecher E Yarnitsky D. CPM test-retest reliability: “standard” vs “single test-stimulus” protocols. Pain Med (United States) 2016;17:521–9. 10.1111/pme.12868 26272736

[24] Harris BS Steiner AZ Jukic AM. Ovarian reserve biomarkers and menstrual cycle length in a prospective cohort study. J Clin Endocrinol Metab 2021;106:e3748–59. 33772306 10.1210/clinem/dgab204 PMC8372629

[25] Hautala AJ Kiviniemi AM Tulppo MP. Individual responses to aerobic exercise: the role of the autonomic nervous system. Neurosci Biobehav Rev 2009;33:107–15. 18514313 10.1016/j.neubiorev.2008.04.009

[26] Hermans L Van Oosterwijck J Goubert D Goudman L Crombez G Calders P Meeus M. Inventory of personal factors influencing conditioned pain modulation in healthy people: a systematic literature review. Pain Pract 2016;16:758–69. 26011523 10.1111/papr.12305

[27] Iacovides S Avidon I Baker FC. Does pain vary across the menstrual cycle? A review. Eur J Pain (UK) 2015;19:1389–405. 10.1002/ejp.714 25899177

[28] Imai Y Petersen KK Mørch CD Arendt Nielsen L. Comparing test–retest reliability and magnitude of conditioned pain modulation using different combinations of test and conditioning stimuli. Somatosensory Mot Res 2016;33:169–77. 10.1080/08990220.2016.1229178 27650216

[29] Ishaque S Khan N Krishnan S. Trends in heart-rate variability signal analysis. Front Digit Health 2021;3:639444. 34713110 10.3389/fdgth.2021.639444 PMC8522021

[30] Jiang M Mieronkoski R Rahmani AM Hagelberg N Salantera S Liljeberg P. Ultra-short-Term analysis of heart rate variability for real-time acute pain monitoring with wearable electronics. Proceedings—2017 IEEE International Conference on Bioinformatics and Biomedicine, BIBM 2017. Institute of Electrical and Electronics Engineers Inc., Vol. 2017. p. 1025–32, 2017.

[31] Kennedy DL Kemp HI Ridout D Yarnitsky D Rice ASC. Reliability of conditioned pain modulation: a systematic review. PAIN 2016;157:2410–9. 27559835 10.1097/j.pain.0000000000000689 PMC5228613

[32] Khasar SG Isenberg WM Miao FJP Gear RW Green PG Levine JD. Gender and gonadal hormone effects on vagal modulation of tonic nociception. J Pain 2001;2:91–100. 14622830 10.1054/jpai.2000.19295

[33] Khasar SG Miao FJP Gear RW Green PG Levine JD. Vagal modulation of bradykinin-induced mechanical hyperalgesia in the female rat. J Pain 2003;4:278–83. 14622697 10.1016/s1526-5900(03)00631-x

[34] Koenig J Jarczok MN Ellis RJ Hillecke TK Thayer JF. Heart rate variability and experimentally induced pain in healthy adults: a systematic review. Eur J Pain (UK) 2014;18:301–14. 10.1002/j.1532-2149.2013.00379.x 23922336

[35] De Kooning M Daenen L Cras P Gidron Y Roussel N Nijs J. Autonomic response to pain in patients with chronic whiplash associated disorders. Pain Physician 2013;16:E277–85. 23703426

[36] Laborde S Mosley E Mertgen A. Vagal tank theory: the three Rs of cardiac vagal control functioning—resting, reactivity, and recovery. Front Neurosci 2018;12:458. 30042653 10.3389/fnins.2018.00458 PMC6048243

[37] Laborde S Mosley E Thayer JF. Heart rate variability and cardiac vagal tone in psychophysiological research—recommendations for experiment planning, data analysis, and data reporting. Front Psychol 2017;8:213–8. 28265249 10.3389/fpsyg.2017.00213 PMC5316555

[38] Li H Gibson EA Jukic AMZ Baird DD Wilcox AJ Curry CL Fischer-Colbrie T Onnela J-P Williams MA Hauser R Coull BA Mahalingaiah S. Menstrual cycle length variation by demographic characteristics from the Apple Women's Health Study. NPJ Digit Med 2023;6:100. 37248288 10.1038/s41746-023-00848-1 PMC10226714

[39] Long J, Interactions: comprehensive, user-friendly toolkit for probing interactions. R package version 1.2.0; 2024. Available at: https://cran.r-project.org/package=interactions. doi:10.32614/CRAN.package.interactions, R package version 1.2.0.

[40] Lovibond PF Lovibond SH. The structure of negative emotional states: comparison of the Depression Anxiety Stress Scales (DASS) with the Beck Depression and Anxiety inventories. Behav Res Ther 1995;33:335–43. 7726811 10.1016/0005-7967(94)00075-u

[41] Makovac E Venezia A Hohenschurz-Schmidt D Dipasquale O Jackson JB Medina S O'Daly O Williams SCR McMahon SB Howard MA Macefield V Scr W. The association between pain‐induced autonomic reactivity and descending pain control is mediated by the periaqueductal grey. J Physiol 2021;599:5243–60. 34647321 10.1113/JP282013

[42] Makowski D Pham T Lau ZJ Brammer JC Lespinasse F Pham H Schölzel C Chen SHA. NeuroKit2: a Python toolbox for neurophysiological signal processing. Behav Res Methods 2021;53:1689–96. 33528817 10.3758/s13428-020-01516-y

[43] Matusik PS Zhong C Matusik PT Alomar O Stein PK. Neuroimaging studies of the neural correlates of heart rate variability: a systematic review. J Clin Med 2023;12:1016. 36769662 10.3390/jcm12031016 PMC9917610

[44] McNeil DW Kennedy SG Randall CL Addicks SH Wright CD Hursey KG Vaglienti R. Fear of Pain Questionnaire‐9: brief assessment of pain‐related fear and anxiety. Eur J Pain 2018;22:39–48. 28758306 10.1002/ejp.1074 PMC5730485

[45] Mertens MG Hermans L Crombez G Goudman L Calders P Van Oosterwijck J Meeus M. Comparison of five conditioned pain modulation paradigms and influencing personal factors in healthy adults. Eur J Pain (UK) 2021;25:243–56. 10.1002/ejp.1665 32965727

[46] Michaeli-Izak E Kodesh E Weissman‐Fogel I. Vagal tone, pain sensitivity and exercise‐induced hypoalgesia: the effect of physical activity level. Eur J Pain 2024;28:1524–35. 38606718 10.1002/ejp.2275

[47] Mihm M Gangooly S Muttukrishna S. The normal menstrual cycle in women. Anim Reprod Sci 2011;124:229–36. 20869180 10.1016/j.anireprosci.2010.08.030

[48] Multon S Schoenen J. Pain control by vagus nerve stimulation: from animal to man and back. Acta Neurol Belg 2005;105:62–7. 16076058

[49] Nahman-Averbuch H Dayan L Sprecher E Hochberg U Brill S Yarnitsky D Jacob G. Sex differences in the relationships between parasympathetic activity and pain modulation. Physiol Behav 2016;154:40–8. 26556539 10.1016/j.physbeh.2015.11.004

[50] Nahman-Averbuch H Nir RR Sprecher E Yarnitsky D. Psychological factors and conditioned pain modulation: a meta-analysis. Clin J Pain 2016;32:541–54. 26340657 10.1097/AJP.0000000000000296

[51] Nahman-Averbuch H Sprecher E Jacob G Yarnitsky D. The relationships between parasympathetic function and pain perception: the role of anxiety. Pain Pract 2016;16:1064–72. 26878998 10.1111/papr.12407

[52] Nahman-Averbuch H Yarnitsky D Granovsky Y Gerber E Dagul P Granot M. The role of stimulation parameters on the conditioned pain modulation response. Scand J Pain 2013;4:10–14. 29913877 10.1016/j.sjpain.2012.08.001

[53] Nahman‐Averbuch H Yarnitsky D Sprecher E Granovsky Y Granot M. Relationship between personality traits and endogenous analgesia: the role of harm avoidance. Pain Pract 2016;16:38–45. 25353647 10.1111/papr.12256

[54] Nijs J De Kooning M Daenen L Cras P Gidron Y Roussel N. Case-Control Study autonomic response to pain in patients with chronic whiplash associated disorders. Pain Physician 2013;16:E277–85. 23703426

[55] Nir R-R Yarnitsky D. Conditioned pain modulation. Top Pain Manag 2015;30:131–8. 10.1097/SPC.0000000000000126 25699686

[56] Nishikawa Y Koyama N Yoshida Y Yokota T. Activation of ascending antinociceptive system by vagal afferent input as revealed in the nucleus ventralis posteromedialis. Brain Res 1999;833:108–11. 10375683 10.1016/s0006-8993(99)01521-8

[57] Ondicova K Pecenak J Mravec B, The role of the vagus nerve in depression, Neuroendocrinol Let. 2010;31:602. 21173739

[58] Pacheco-Barrios K Gianlorenco AC Camargo L Andrade MF Choi H Song JJ Fregni F. Transauricular Vagus Nerve Stimulation (taVNS) enhances Conditioned Pain Modulation (CPM) in healthy subjects: a randomized controlled trial. Brain Stimulation. 2024;17:346–8. 38453004 10.1016/j.brs.2024.03.006

[59] Porges SW. Polyvagal theory: a science of safety. Front Integr Neurosci 2022;16:871227. 35645742 10.3389/fnint.2022.871227 PMC9131189

[60] Porges SW. The polyvagal perspective. Biol Psychol 2007;74:116–43. 17049418 10.1016/j.biopsycho.2006.06.009 PMC1868418

[61] Ramaswamy S Wodehouse T. Conditioned pain modulation—a comprehensive review. Neurophysiol Clin 2021;51:197–208. 33334645 10.1016/j.neucli.2020.11.002

[62] Randich A Gebhart GF. Vagal afferent modulation of nociception. Brain Res Rev 1992;17:77–99. 1327371 10.1016/0165-0173(92)90009-b

[63] Rezaii T Hirschberg AL Carlström K Ernberg M. The influence of menstrual phases on pain modulation in healthy women. J Pain 2012;13:646–55. 22634142 10.1016/j.jpain.2012.04.002

[64] Schmalenberger KM Eisenlohr-Moul TA Jarczok MN Eckstein M Schneider E Brenner IG Duffy K Schweizer S Kiesner J Thayer JF Ditzen B. Menstrual cycle changes in vagally-mediated heart rate variability are associated with progesterone: evidence from two within-person studies. J Clin Med 2020;9:617. 32106458 10.3390/jcm9030617 PMC7141121

[65] Schmalenberger KM Eisenlohr-Moul TA Würth L Schneider E Thayer JF Ditzen B Jarczok MN. A systematic review and meta-analysis of within-person changes in cardiac vagal activity across the menstrual cycle: implications for female health and future studies. J Clin Med 2019;8:1946. 31726666 10.3390/jcm8111946 PMC6912442

[66] Schmalenberger KM Tauseef HA Barone JC Owens SA Lieberman L Jarczok MN Girdler SS Kiesner J Ditzen B Eisenlohr-Moul TA. How to study the menstrual cycle: practical tools and recommendations. Psychoneuroendocrinology 2021;123:104895. 33113391 10.1016/j.psyneuen.2020.104895 PMC8363181

[67] Sgoifo A Carnevali L Pico Alfonso MDLA Amore M. Autonomic dysfunction and heart rate variability in depression. Stress 2015;18:343–52. 26004818 10.3109/10253890.2015.1045868

[68] Shaffer F Meehan ZM Zerr CL. A critical review of ultra-short-term heart rate variability norms research. Front Neurosci 2020;14:594880. 33328866 10.3389/fnins.2020.594880 PMC7710683

[69] Suarez‐Roca H Mamoun N Sigurdson MI Maixner W. Baroreceptor modulation of the cardiovascular system, pain, consciousness, and cognition. Compr Physiol 2021;11:1373–423. 33577130 10.1002/cphy.c190038 PMC8480547

[70] Suarez-Roca H Mamoun N Watkins LL Bortsov AV Mathew JP. Higher cardiovagal baroreflex sensitivity predicts increased pain outcomes after cardiothoracic surgery. J Pain 2024;25:187–201. 37567546 10.1016/j.jpain.2023.08.002 PMC10841280

[71] Sullivan MJL Bishop SR Pivik J. The pain catastrophizing scale: development and validation. Psychol Assess 1995;7:524–32.

[72] Sun Z. Cardiovascular responses to cold exposure. Front Biosci 2010;E2:495–503. 10.2741/e108 PMC2826836 20036896

[73] Thayer JF Hansen AL Saus-Rose E Johnsen BH. Heart rate variability, prefrontal neural function, and cognitive performance: the neurovisceral integration perspective on self-regulation, adaptation, and health. Ann Behav Med 2009;37:141–53. 19424767 10.1007/s12160-009-9101-z

[74] Thayer JF Lane RD. Claude Bernard and the heart–brain connection: further elaboration of a model of neurovisceral integration. Neurosci Biobehav Rev 2009;33:81–8. 18771686 10.1016/j.neubiorev.2008.08.004

[75] Tousignant-Laflamme Y Marchand S. Excitatory and inhibitory pain mechanisms during the menstrual cycle in healthy women. PAIN 2009;146:47–55. 19592167 10.1016/j.pain.2009.06.018

[76] Tracy LM Jarczok MN Ellis RJ Bach C Hillecke TK Thayer JF Koenig J. Heart rate variability and sensitivity to experimentally induced pain: a replication. Pain Pract 2018;18:687–9. 29194925 10.1111/papr.12652

[77] Traxler J Hanssen MM Lautenbacher S Ottawa F Peters ML. General versus pain‐specific cognitions: pain catastrophizing but not optimism influences conditioned pain modulation. Eur J Pain 2019;23:150–9. 30074678 10.1002/ejp.1294 PMC6585813

[78] Uzawa H Takeuch S Nishida Y. Sex differences in conditioned pain modulation effects and its associations with autonomic nervous system activities in healthy, younger individuals: a pilot study. Pain Rep 2024;9:E1123. 38322355 10.1097/PR9.0000000000001123 PMC10843308

[79] Vollert J Trewartha N Kemkowski D Cremer AF Zahn PK Segelcke D Pogatzki-Zahn EM. Conditioned pain modulation and offset analgesia: influence of sex, sex hormone levels and menstrual cycle on the magnitude and retest reliability in healthy participants. Eur J Pain (UK) 2022;26:1938–49. 10.1002/ejp.2014 35856832

[80] Weissman-Fogel I Dror A Defrin R. Temporal and spatial aspects of experimental tonic pain: understanding pain adaptation and intensification. Eur J Pain (UK) 2015;19:408–18. 10.1002/ejp.562 25045086

[81] Wilson H Carvalho B Granot M Landau R. Temporal stability of conditioned pain modulation in healthy women over four menstrual cycles at the follicular and luteal phases. PAIN 2013;154:2633–8. 23811040 10.1016/j.pain.2013.06.038

[82] Yan XJ Feng CC Liu Q Zhang LY Dong X Liu ZL Cao ZJ Mo JZ Li Y Fang JY Chen SL. Vagal afferents mediate antinociception of estrogen in a rat model of visceral pain: the involvement of intestinal mucosal mast cells and 5-hydroxytryptamine 3 signaling. J Pain 2014;15:204–17. 24231720 10.1016/j.jpain.2013.10.012

[83] Yarnitsky D Bouhassira D Drewes AM Fillingim RB Granot M Hansson P Landau R Marchand S Matre D Nilsen KB Stubhaug A Treede RD Wilder-Smith OHG. Recommendations on practice of conditioned pain modulation (CPM) testing. Eur J Pain (UK) 2015;19:805–6. 10.1002/ejp.605 25330039

[84] Yuan H Silberstein SD. Vagus nerve and vagus nerve stimulation, a comprehensive review: part III. Headache 2016;56:479–90. 26364805 10.1111/head.12649

[85] Zhang Y Luo Y Wu Q Han M Wang H Kang F. Effect of transcutaneous auricular vagus nerve stimulation on conditioned pain modulation in trigeminal neuralgia patients. Pain Ther 2024;13:1529–40. 39259413 10.1007/s40122-024-00654-x PMC11543976

